# The impact of informing diagnosis on quality of life in patients with cancer: A protocol of systematic review and meta-analysis

**DOI:** 10.1097/MD.0000000000012320

**Published:** 2018-09-14

**Authors:** Yabin Li, Miao Wan, Xianggui Luo, Jiao Li, Hongxia Wang, Dang Wei, Haixia Feng

**Affiliations:** a Department of Neurological Rehabilitation, Rehabilitation Central Hospital of Gansu Province; b The First Clinical Medical College of Lanzhou University, Lanzhou, China; c Department of Public Health Sciences, Karolinska Institutet, Stockholm, Sweden.

**Keywords:** awareness of diagnosis, cancer, disclosure of diagnosis, meta-analysis, quality of life, systematic review

## Abstract

**Background:**

Cancer is the second leading cause of death globally. More millions new cancer cases are diagnosed, and millions persons died due to cancer each year. There are different attitudes on disclosure of diagnosis to the patients with cancer. The current systematic reviews are qualitative, and indicate that there is limited evidence on the association between awareness of diagnosis and quality of life in patients with cancer. In this study, we aim to evaluate the effect of awareness of diagnosis on quality of life in patients with cancer by conducting a systematic review and meta-analysis.

**Methods:**

We will perform a comprehensive electronic search in the databases below: MEDLINE (via PubMed), EMBASE, Cochrane Central Register of Controlled Trials, PsycINFO, WEB OF SCIENCE, Chinese Biomedical Literature database, WANFANG database, and China National Knowledge Infrastructure. The cohort studies focusing on the association between awareness of diagnosis and quality of life in patients with cancer will be included. The risk of bias for the included studies will be appraised using the Newcastle-Ottawa Scale tool for cohort study. We will pool the effect estimates from the included studies to quantitatively present the strength of the association of interest.

**Results:**

This study will present pooled effect estimates regarding the impact of informing diagnosis on quality of life in patients with cancer.

**Conclusion:**

This is the first quantitative systematic review which tends to provide modest evidence on the association between informing diagnosis and quality of life in patients with cacner.

**PROSPERO registration number:**

CRD42017060073.

## Abstract 2

Supplemental Digital Content is available in the text

## 1 Background

In 2015, about 17.5 million incident cancer cases were diagnosed worldwide, with increasing by 33% from 2005 to 2015, and about 8.7 million persons died due to cancer.^[1]^ With regard to the perspective of disclosing the diagnosis of cancer, it varies in different cultures and in different populations such as physician and family member.^[2]^ In Middle East regions, the physicians follow a truth disclosure policy, which they inform 1 of the family members about the diagnosis for respecting the traditional culture and tell the patients the truth when possible, whereas the family members are more likely to conceal the truth to their relatives with cancer.^[3]^ Although many patients with cancer can live longer than before as a consequence of the improvement of health care and treatment for cancer, most of these patients die due to cancer several years after diagnosing. The perception of cancer's incurability together with other factors such as cultural background, personal preference, age, sex, and so on contribute to concealment of diagnosis of cancer in many countries.^[4]^ For the patients with cancer, 2 previous systematic reviews indicate that there is limited evidence to confirm the influence of awareness of diagnosis on quality of life.^[5,6]^ In the 2 systematic reviews, the quantitative method such as meta-analysis had not been applied, which might not be helpful for the evidence end-users to understand the association between disclosure of diagnosis and quality of life. Fortunately, a range of quantitative studies focusing on this topic has been published to date.^[7–10]^ However, there is no systematic review with meta-analysis to assess the effect of awareness of diagnosis on quality of life in patients with cancer.

In this review, we aim to systematically collect and review the studies focusing on the association between disclosure of diagnosis of cancer and quality of life, and conduct mate-analysis to quantitatively present the association by pooling the effect estimates.

## 2 Methods

To produce a high-quality systematic review and meta-analysis, we conduct and do this research rigidly complying with the methods from the Cochrane Collaboration and the reporting guideline. Thus, the protocol of this study has been registered on the PROSPERO international prospective register of systematic reviews (Register number: CRD42017060073). The reporting of this protocol was in accordant with the Preferred Reporting Items for Systematic Review and Meta-analysis Protocol.^[11,12]^

### 2.1 Eligibility criteria

We will screen the eligible studies according to the following criteria: the patients with diagnosis of any type of cancer; aim to evaluate whether being aware of the diagnosis of cancer influence the quality of life compared with those patients without awareness of diagnosis; reported the outcome of quality of life measured by European Organization for Research and Treatment of Cancer (EORTC) Quality of Life Questionnaire (EORTC QLQ-C30), Functional Assessment of Cancer Therapy, McGill Quality of Life Questionnaire, City of Hope Quality of Life Questionnaire, Supportive Care Needs Survey, 36-Item Short Form Health Survey, Quality of Life in Adult Cancer Survivors, and so on; cohort study (initially, the relevant systematic reviews and meta-analyses will also be included for tracking their references); published in English and Chinese. We will exclude the studies of which only the conference abstracts were published or the full texts were not available after contacting the correspondence author.

### 2.2 Information source

We will develop a comprehensive search strategy led by an author, Dang Wei (DW), who is professional in information search and systematic review. The databases we plan to search include: MEDLINE (via PubMed), Cochrane Central Register of Controlled Trials, PsycINFO, WEB OF SCIENCE, EMBASE, Chinese Biomedical Literature database, Chinese Medicine Premier (WANFANG database), and China National Knowledge Infrastructure.

In addition, after screening the records retrieving from the databases, we will check the references of the eligible studies to obtain further relevant studies.

### 2.3 Search strategy

We will perform the electronic search in the above databases from the inception of databases to August, 2018 and without any language limitation. The search terms in the search strategy comprise the academic terms regarding cancer, awareness of diagnosis, and quality of life. The search strategy presented below is the details of search what we will perform in PubMed:

1. #1 “neoplasms ” [Mesh] OR cancer[Title/Abstract] OR malignancy [Title/Abstract] OR tumour[Title/Abstract] OR tumor[Title/Abstract] OR neoplasm[Title/Abstract] OR carcinoma[Title/Abstract]
2. #2 disclosure [Title/Abstract] OR truth telling [Title/Abstract] OR breaking bad news[Title/Abstract] OR knowledge[Title/Abstract] OR knowing[Title/Abstract] OR awareness[Title/Abstract]
3. #3 quality of life[Title/Abstract] OR QOL[Title/Abstract]
4. #4 #1 AND #2 AND #3

The more details of search strategy of this systematic review can be found in the supplement file.

### 2.4 Study selection and data extraction

After performing the electronic search, the records retrieved will be imported to EndNote X7 literature management software. Then, pairs of authors will firstly screen for the potentially eligible studies independently by reading the title and abstract, and review the full texts of the potential eligible studies further. In the full-text reviewing stage, we will record each study excluded and the reasons for exclusion.

Pairs of reviewers independently extracted the data of included studies. The items we plan to extract include: basic characteristics (title, first author, publication year, country, journal, financial support, conflicts of interest, etc), study design (the setting where the research was carried out, the time period when the study was performed, the length of follow up, the definition of exposure on the diagnosis of cancer, etc), participant data (number of patients in each group, type of cancer, tumor stage, co-morbidities, lost/withdrawal, etc), and outcome (measured tools, time points, and results, etc).

We plan to do a pilot test for each assignment, respectively, to ensure high inter-rater reliability among the researchers before the normal study screening and data extraction. When meeting disagreements, discussion will be organized and the conflicts will be solved by a third researcher (DW).

### 2.5 Risk of bias assessment

Although the risk of bias tool for nonrandomized studies of intervention has been published,^[13]^ the Newcastle-Ottawa Scale (NOS) for cohort study seems more feasible to assess the risk of bias of cohort study.^[14]^ Thus, we will use the NOS tool to assess the risk of bias of the included studies in this systematic review. The tool comprises 8 items which are representativeness of the exposed cohort, selection of nonexposed cohort, ascertainment of exposure, demonstration that outcome of interest was not present at start of study, comparability of cohorts on the basis of the design or analysis controlled for confounders, assessment of outcome, was follow-up long enough for outcomes to occur, and adequacy of follow-up of cohorts. The included studies will be assessed as good, fair, and poor quality according to the number of items which the studies meet. When meeting disagreement, discussion will be organized or solved by DW.

### 2.6 Data synthesis

In this systematic review, the outcomes related to quality of life were measured by the scales of which the results are continuous. Thus, we will calculate the standardized mean differences (SMDs) with 95% confidence intervals for assessing the association between awareness of diagnosis of cancer and quality of life in patients with cancer. The pooled SMDs will be obtained by performing meta-analysis in STATA V.12.0. Before performing data synthesis, we plan to evaluate the heterogeneity by I^2^ statistics. If I^2^ ≤50%, the Mantel-Haenszel fixed-effects model will be applied to pool the data. Otherwise, we will do subgroup analysis and meta-regression to test the sources of heterogeneity before deciding whether synthesizing the data or not. If there is no evidence on clinical heterogeneity, the data synthesis will be performed by the Mantel-Haenszel random-effects model. But if it indicates clear evidence on clinical heterogeneity, we will conduct subgroup analysis (if power is enough); otherwise, we will only describe the results of the included studies, respectively.

For the assessment of reporting bias, we plan to produce the funnel plot by Begg and Egger method,^[15,16]^ and judge the publication bias through visual analysis of funnel plots initially. In addition, the contour-enhanced funnel plot will be used to assist to distinguish asymmetry, if multifactors lead to publication bias.^[17]^

### 2.7 Quality of evidence

We will generate the summary of findings tables using the GRADEpro—GDT system (https://gradepro.org/). For each outcome, the quality of the evidence will be assessed by a pair of authors (DW, YL) independently, and the results will be discussed in a research group meeting. Firstly, according to the Grading of Recommendation, Assessment, Development, and Evaluation approach (GRADE),^[18,19]^ we will assess the following aspects of the bodies of evidence to decide whether the quality of evidence will be downgraded or not: risk of bias, directness, heterogeneity, precision of effect estimates, and publication bias. When the quality of evidence is not downgraded in the 5 domains, we will evaluate further whether the quality of evidence would be upgraded by the following items: large magnitude of effect, dose-response gradient and plausible confounding, because the quality of evidence from the observed studies is rated as low primarily. If the quality of evidence has been downgraded, it will not be considered to upgrade. Finally, the quality of evidence for each outcome would be rated as “high.” “moderate,” “low,” or “very low.”

### 2.8 Ethics and dissemination

This research is a systematic review and network meta-analysis. Thus, there is no requirement of ethical approval and patient informed consent.

## 3 Discussion

Even though there are several systematic reviews on the association between awareness of diagnosis and quality of life in patients with cancer,^[5,6]^ any quantitative results have been presented. This review is the first study which tries to systematically retrieve and review the current relevant primary studies on the association and synthesize the effect estimates from the included studies. Moreover, in this study, we plan to use GRADE approach to assess the quality of evidence, which would present how much confidence we have on the findings. Eventually, this review would be likely to well inform the policy and decision making on healthcare for the patients with cancer in the public or clinical practice.

## Author contributions

Contributors: Conception and design of this systematic review and Bayesian network meta-analysis (Yabin Li, Miao Wan, Xianggui Luo, Dang Wei, Haixia Feng); tested the feasibility of the study (Yabin Li, Miao Wan, Xianggui Luo, Jiao Li, Hongxia Wang); developed the search strategy (Dang Wei, Miao Wan, Xianggui Luo); drafted this protocol (Yabin Li, Miao Wan, Xianggui Luo, Haixia Feng), revised the protocol (Yabin Li, Miao Wan, Xianggui Luo, Dang Wei, Jiao Li, Hongxia Wang). All authors provided critical revisions of the protocol and approved the final manuscript.

**Conceptualization:** Yabin Li, Miao Wan, Xianggui Luo, Dang Wei, Haixia Feng.

**Investigation:** Jiao Li, Hongxia Wang.

**Methodology:** Miao Wan, Xianggui Luo, Dang Wei.

**Supervision:** Haixia Feng.

**Writing – original draft:** Yabin Li, Miao Wan, Xianggui Luo, Haixia Feng.

**Writing – review & editing:** Yabin Li, Miao Wan, Xianggui Luo, Jiao Li, Hongxia Wang, Dang Wei.

Dang Wei orcid: 0000-0003-1287-7929

## Supplementary Material

Supplemental Digital Content
