# Supplementary material for: The impact of informing diagnosis on quality of life in patients with cancer: A protocol of systematic review and meta-analysis
Source: Medicine (Baltimore). 2018 Sep 14;97(37):e12320. doi: 10.1097/MD.0000000000012320 (PMC6155951; doi:10.1097/MD.0000000000012320)
Supplement: Supplemental Digital Content [file medi-97-e12320-s001.pdf]

Search strategy

Pubmed

#1 "neoplasms " [Mesh]
#2 cancer[Title/Abstract]
#3 malignancy [Title/Abstract]
#4 tumour[Title/Abstract]
#5 tumor[Title/Abstract]
#6 neoplasm[Title/Abstract]
#7 carcinoma[Title/Abstract]
#8 #1 OR #2 OR #3 OR #4 OR #5 OR #6 OR #7
#9 disclosure [Title/Abstract]
#10 truth telling [Title/Abstract]
#11 breaking bad news[Title/Abstract]
#12 knowledge[Title/Abstract]
#13 knowing[Title/Abstract]
#14 awareness[Title/Abstract]
#15 #9 OR #10 OR #11OR #12 OR #13 OR #14
#16 quality of life[Title/Abstract]
#17 QOL[Title/Abstract]
#18 #16 OR #17
#19 #8 AND #15 AND #18

Embase

#1 "neoplasms "/exp
#2 cancer:ab,ti
#3 malignancy:ab,ti
#4 tumour:ab,ti
#5 tumor:ab,ti
#6 neoplasm:ab,ti
#7 carcinoma:ab,ti
#8 #1 OR #2 OR #3 OR #4 OR #5 OR #6 OR #7
#9 disclosure :ab,ti
#10 truth AND telling:ab,ti
#11 breaking AND bad AND news:ab,ti
#12 knowledge:ab,ti
#13 knowing:ab,ti
#14 awareness:ab,ti
#15 #9 OR #10 OR #11OR #12 OR #13 OR #14
#16 quality AND of AND life:ab,ti
#17 QOL:ab,ti
#18 #16 OR #17

#19 #8 AND #15 AND #18
#20 #19 AND [embase]/lim NOT [medline]/lim

CENTRAL
#1 Mesh descriptor: [neoplasms] explode all trees
#2 cancer or malignancy or tumour or tumor or neoplasm or carcinoma:ti,ab,kw (Word variations have been searched)
#3 disclosure or truth telling or breaking bad news or knowledge or knowing or awareness :ti,ab,kw (Word variations have been searched)
#4 quality of life or QOL:ti,ab,kw (Word variations have been searched)
#5 #1 or #2
#6 #3 and #4 and #5

PsycINFO
#1 AB,TI(cancer)
#2 AB,TI(malignancy)
#3 AB,TI(tumour)
#4 AB,TI(tumor)
#5 AB,TI(neoplasm)
#6 AB,TI(carcinoma)
#7 #1 OR #2 OR #3 OR #4 OR #5 OR #6
#8 AB,TI(disclosure)
#9 AB,TI(truth telling)
#10 AB,TI(breaking bad news)
#11 AB,TI(knowledge)
#12 AB,TI(knowing)
#13 AB,TI(awareness)
#14 #8 OR #9 OR #10 OR #11OR #12 OR #13
#15 AB,TI(quality of life)
#16 AB,TI(QOL)
#17 #15 OR #16
#18 #7 AND #14 AND #17

WEB OF SCIENCE
#1 topic:(cancer) OR topic:(malignancy) OR topic:(tumour) OR topic:(tumor) OR topic:(neoplasm) OR topic:(carcinoma)
#2 topic:(disclosure) OR topic:(truth telling) OR topic:(breaking bad news) OR topic:(knowledge) OR topic:(knowing) OR topic:(awareness)
#3 topic:(quality of life) OR topic:(QOL)
#4 #3AND#2 AND#1

CBM

#1 缺省[智能]:癌
#2 缺省[智能]:瘤
#3 主题词:肿瘤/全部树/全部副主题词
#4 缺省[智能]:生活质量
#5 缺省[智能]:知晓
#6 缺省[智能]:告知
#7 缺省[智能]:知情
#8 #1 or #2 or #3
#9 #5 or #6 or #7
#10 #4 and #8 and #9

WANFANG database

主题:(癌 + 瘤) * 主题:(生活质量) * 主题:(知晓 + 告知 + 知情)

CNKI

#1 主题=癌
#2 主题=瘤
#3 #1 or #2
#4 主题=知晓
#5 主题=知情
#6 主题=告知
#7 #4 or #5 or #6
#8 主题=生活质量
#9 #3 and #7 and #8
